# Preparation and Performance of a Low-Carbon Foam Material of Fly-Ash-Based Foamed Geopolymer for the Goaf Filling

**DOI:** 10.3390/ma13040841

**Published:** 2020-02-12

**Authors:** Lijuan Su, Guosheng Fu, Yunlong Wang, Guangchun Yao, Jianing Zhang, Xinchao Xu, Baoxin Jia

**Affiliations:** 1 School of Civil Engineering, Liaoning Technical University, Fuxin 123000, China; fuguosheng1004@163.com (G.F.); sulijuan87@163.com (Y.W.); zhangjianing0219@163.com (J.Z.); xuxinchao84@163.com (X.X.); jiabaoxin@lntu.edu.cn (B.J.); 2 School of metallurgy, Northeastern University, Shenyang 110819, China; su_ljd@163.com

**Keywords:** foamed geopolymer, fly ash, uniaxial compression, microstructure, stress-strain curve

## Abstract

The treatment of goaf subsidence is important for sustainable development. Geopolymer is a new type of cementing material with excellent mechanical properties, durability, corrosion resistance, and other advantages owing to its unique three-dimensional spatial aggregation structure. Herein, a type of preparation technology of fly-ash-based foamed geopolymer suitable for goaf filling was developed by adding a chemical foaming agent to the matrix of fly-ash-based geopolymer. The mechanical properties, chemical composition, and pore structure characteristics of the samples were discussed. When the samples with different contents of fly ash, sodium metasilicate, sodium stearate, H_2_O_2_, and NaOH were prepared, a uniaxial compression test was performed to analyze the uniaxial compression failure characteristics and compression strength of the samples. The mineralogical composition of each sample was analyzed by X-ray diffraction (XRD) test, and the microstructure images of different samples were observed using scanning electron microscopy (SEM). The effects of the content of each component on the properties of the samples were discussed. Finally, the CO_2_ emission, energy consumption, and cost of producing fly-ash-based foamed geopolymer were analyzed. Overall, the material had the advantages of low energy consumption, low CO_2_ emission, environmental-protection ability, and waste utilization and thus has a broad application prospect in treating subsidence.

## 1. Introduction

With the large-scale exploitation of mineral resources, the problem of goaf subsidence has increasingly become prominent [1,2]. If untreated, then it causes instability of the mountain as well as tilting of the surrounding buildings, crack, and other damages, thereby resulting in large-scale security risks. At the same time, the collapse of the mined out area causes serious damage to the surface ecological environment and a series of geological environmental problems, such as large-scale water accumulation and surface cracking, thereby resulting in great difficulty to the subsequent geological environmental treatment and ecological restoration [3]. This finding is contrary to the introduction of the basic strategy of “insisting on the harmonious coexistence of human and nature” by the Chinese government. In addition, this result does not conform to the Chinese government’s concept of sustainable development and green production. Therefore, developing a green and low carbon filling material for goaf filling is urgent.

At present, researchers tend to use foamed concrete as filling material [4,5,6,7,8,9]. Although this material is feasible from the perspective of bearing capacity, the cement production process needs to go through a high-temperature calcining procedure that consumes substantial resources and energy and requires limestone raw materials, resulting in a large amount of CO_2_ emissions [10,11,12]. Thus, using foamed concrete as goaf filling material is harmful to the ecological environment [13,14]. Geopolymer is a new type of cementitious material, which has the advantages of fast condensation and hardening, low hydration heat, small shrinkage, good impermeability and frost resistance, corrosion resistance, and high temperature resistance. Most importantly, its production process is pollution-free and belongs to low CO_2_ emission materials [15,16,17,18,19,20,21]. Apart from being used as civil engineering materials, they can be used to seal toxic and harmful heavy metals and produce high-temperature fireproof materials and new light insulation materials [22,23,24,25,26,27,28].

At the same time, with the development of the power industry, the fly ash emission of coal-fired power plants annually increases and has become one of the most widespread industrial wastes with large emissions in China [29,30,31,32,33,34,35,36]. If a large amount of fly ash remains untreated, then dust and pollution become serious; if it is discharged into the water system, then it causes river silting, and the toxic chemicals it possesses harm the human body and organism [37,38,39]. With the development of the country, the demand for electric power continues to grow. Therefore, the output of fly ash in China is predicted to continuously grow in the future, reaching 925 million tons by 2024. However, compared with developed countries, the comprehensive utilization rate of fly ash in China is still relatively low [40,41].

Recently, fly ash has been used to synthesize geopolymer at room temperature after mechanical activation for 120 min [42]. The relationship among the grinding process of fly ash, the characteristics of ground material and the characteristics of geopolymer has been established [43]. The effects of the fly ash fineness on the rheology of geopolymer paste and the foam properties have also been investigated [44]. This study aimed to develop a type of foamed geopolymer material for goaf filling. Samples with different contents of sodium metasilicate, NaOH, H_2_O_2_, and sodium stearate were prepared, and their physical properties, mechanical properties, mineral composition, microstructure, cost, and energy consumption were analyzed and discussed.

## 2. Experiment

### 2.1. Material

The class F fly ash collected by the electrostatic sedimentation method in Fuxin thermal power plant (Fuxin, China) is used. According to the classification of fly ash in ASTM C618-17 [45], the CaO content in the fly ash is 1.84%, less than 5%; it belongs to low calcium fly ash, i.e., class F fly ash. P·O42.5 ordinary Portland cement produced by Fuxin Daying Cement Manufacturing Co., Ltd (Fuxin, China) is selected. Strong alkaline activator is used to activate the activity of fly ash, which primarily including NaOH and sodium metasilicate, analytically pure NaOH produced by Shenyang Huadong reagent factory (Shenyang, China), and analytically pure sodium metasilicate (white powder) produced by Shenyang Huadong reagent factory. Hydrogen peroxide with volume concentration of 30% is used as foaming agent. The analytical pure sodium stearate produced by Tianjin Zhiyuan Chemical Reagent Co., Ltd. (Tianjin, China) is used as foam stabilizer, which is white fine powder. The water used in the experiment is ordinary tap water.

### 2.2. Experimental Design

On the basis of previous results [11], the dry density of foamed material used for goaf filling was approximately 200–600 kg/m^3^. In this experiment, the foamed geopolymer samples with the target dry density of 420 kg/m^3^, cement content of 0–30%, and liquid–solid ratio of 0.43 were prepared. To study the influence of the content of different components on the properties of foamed geopolymers, samples with different mix proportions are designed according to Table 1.

### 2.3. Manufacturing Process of Foamed Geopolymer

**Figure 1:**
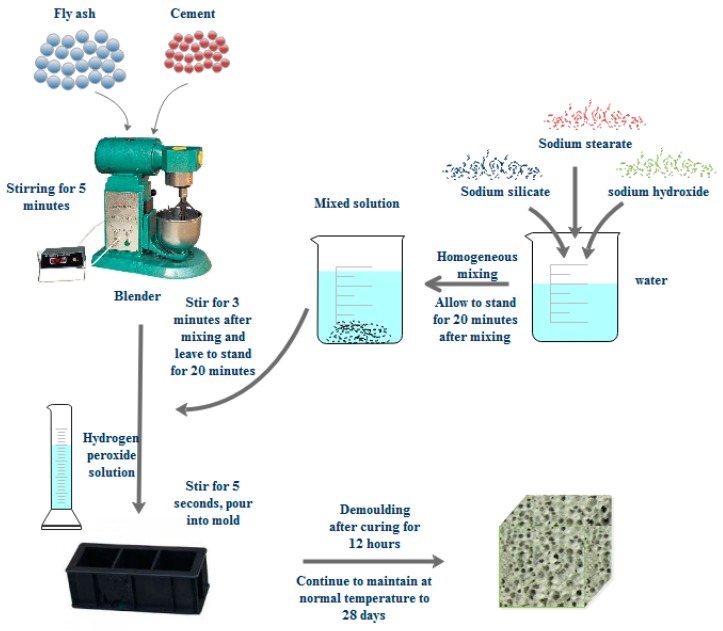
Manufacturing process of fly-ash-based foamed geopolymer.

The cement, fly ash, sodium metasilicate, water, NaOH, and sodium stearate are weighed in accordance with the experimental design ratio. The entire preparation process is divided into four steps, namely, dry material mixing, activator configuration, foaming, pouring into the mold and curing (Figure 1).

Dry material mixing, fly ash and cement should be pre-treated prior to use and placed into the oven for drying at 80 °C for 2 h to avoid moisture of raw materials. Fly ash and cement are poured into a cement mortar mixer and stirred for 5 min to mix evenly.

Activator configuration, sodium metasilicate, NaOH, and sodium stearate are poured into 40 °C water, stirred for 2 min to fully dissolve, and then mixed evenly and set for 20 min.

Foaming: the activator solution is poured into the cement mortar mixer and mixed with the dry materials, stirred for 3 min, mixed evenly, and set for 20 min, thereby allowing the activator solution to fully react with the fly ash in the dry materials.

Pouring into the mold and curing: the hydrogen peroxide solution is poured into the mixer and rapidly stirred for 5 s. Then, the mixed slurry is poured into the 100 mm × 100 mm × 100 mm mold prepared in advance, set for 24 h prior to removing the mold, and finally placed into the constant temperature and humidity curing box for 28 d to obtain the required fly-ash-based foamed geopolymer cube sample. Then, the performance test is conducted.

### 2.4. Characterization of Fly-Ash-Based Foamed Geopolymer

#### 2.4.1. X-ray Diffraction (XRD)

The phase composition of the sample was analyzed with a D8 advance X-ray diffractometer of Bruker Company. The X-ray diffractometer was D/max-γ B X-ray diffractometer, the radiation source was Cu Target Kα, the working voltage was 40 kV, current is 30 mA, and the wavelength was 1.5406 Å. The crystal structure and relative content were analyzed by scanning from 5° to 80° at 10°/min.

#### 2.4.2. Microscopy Appearance

The micro-morphology of the foamed geopolymer was observed using a FEI Quanta 250 FEG scanning electron microscope. The magnification was between 25 and 4000.

#### 2.4.3. Compression Performance

WDW-300E electronic universal testing machine of Jinan Times Gold Testing Machine Co., Ltd. (Jinan, China) was used to test the uniaxial compression performance of the samples to compare the uniaxial compressive strength of the samples with different mix proportions. The loading mode is strain controlled, and the loading rate is 0.2 mm/min. The compressive strength of the sample is obtained as follows:

(1) $$\sigma_{c}=\frac{F_{\max}}{A}$$

where $\sigma_{c}$ is the compressive strength of the material, $F_{\max}$ is the maximum load during the loading process, and A is the area of the sample.

#### 2.4.4. Density

The average density of the three samples of the same mix proportion is obtained using the following calculation formula:

(2) $$\rho =\frac{M}{V}$$

where ρ is the density of the sample, M is the mass of the sample, and V is the volume of the sample.

## 3. Results and Discussion

**Figure 2:**
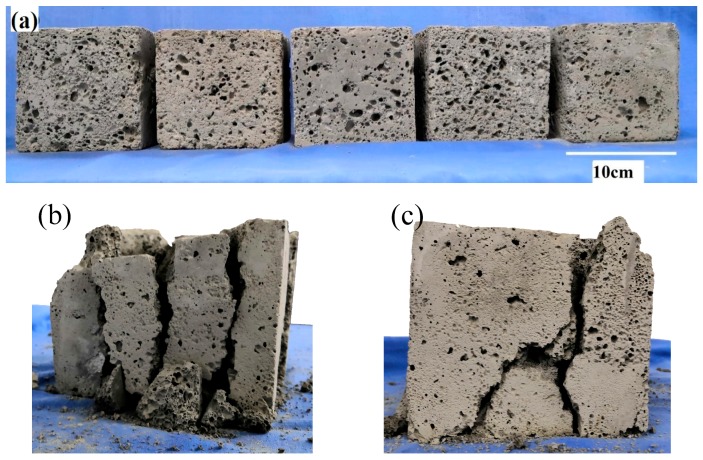
Samples of fly-ash-based foamed geopolymer, (**a**) samples before experiment, (**b**) sample with vertical cracks and (**c**) sample with oblique cracks.

The foamed geopolymer samples are rough, lusterless, and gray. The pore size of the sample surface is different, the distribution is irregular, and the pore size range is 1–8 mm. Figure 2a shows the sample in this experiment.

### 3.1. Physical and Mechanical Properties

Uniaxial compression test was carried out to obtain the compressive strength and stress–strain curve of each sample.

#### 3.1.1. Characteristics of Compression Failure

During the uniaxial compression experiment, the failure characteristics of the samples were observed (Figure 2a). The findings indicate that one or more cracks appear at the end of the sample in the loading process. The crack gradually expands with increased load, and the propagation direction differs for each sample, primarily including vertical crack (Figure 2b) and oblique crack (Figure 2c). Some samples have several cracks in the beginning (Figure 2c). As the cracks expand, the oblique cracks gradually intersect with the vertical cracks and merge into a main crack. Then, the main crack gradually penetrates along the direction of the compression load, and the sample is split into several parts. Some samples show several vertical cracks in the beginning (Figure 2b). The cracks gradually expand with increased load. After penetrating along the direction of the load, the samples are divided into several vertical cracks.

**Figure 3:**
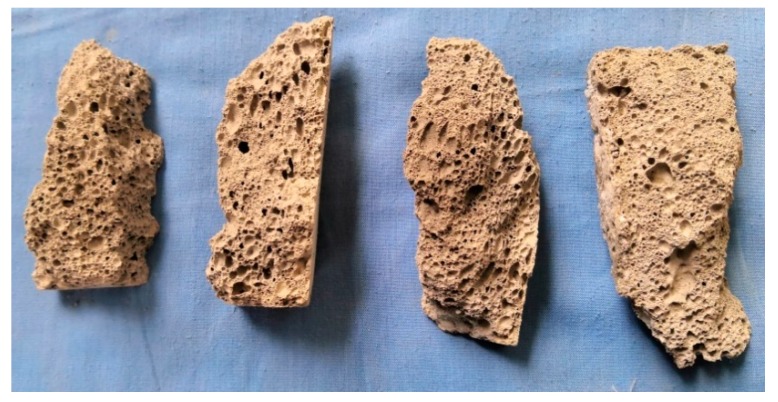
Compression failure characteristics of fly-ash-based foamed geopolymer.

The internal section of the sample after failure was observed. The findings indicate that most internal pore structures of the sample are closed, with different pore sizes, irregular distribution of pore structures, and have no orientation (Figure 3). Part of the large hole is fused with the surrounding holes in the manufacturing process, forming a “local large hole.” The vertical bar after the failure of the test piece shows that a fracture surface exists in the part, where the large hole is concentrated. The vertical bar after the sample is damaged shows that a continuous fracture surface exists at the place, where the large hole is concentrated. Some pore structures have initial defects, such as holes in the hole wall, cracks on the hole edge or hole wall, and defects on the hole wall. These initial defects form an invisible “weak zone” or “weak surface” in the inner part of the test sample, which provides a rapid channel for the penetration of external cracks and accelerates the failure process of the test block.

This analysis shows that the failure of fly-ash-based foamed geopolymer is primarily related to the “local large hole” in the sample and the bearing “weak zone” or “weak surface” formed by various initial defects.

#### 3.1.2. Stress-Strain Curve of Uniaxial Compression

**Figure 4:**
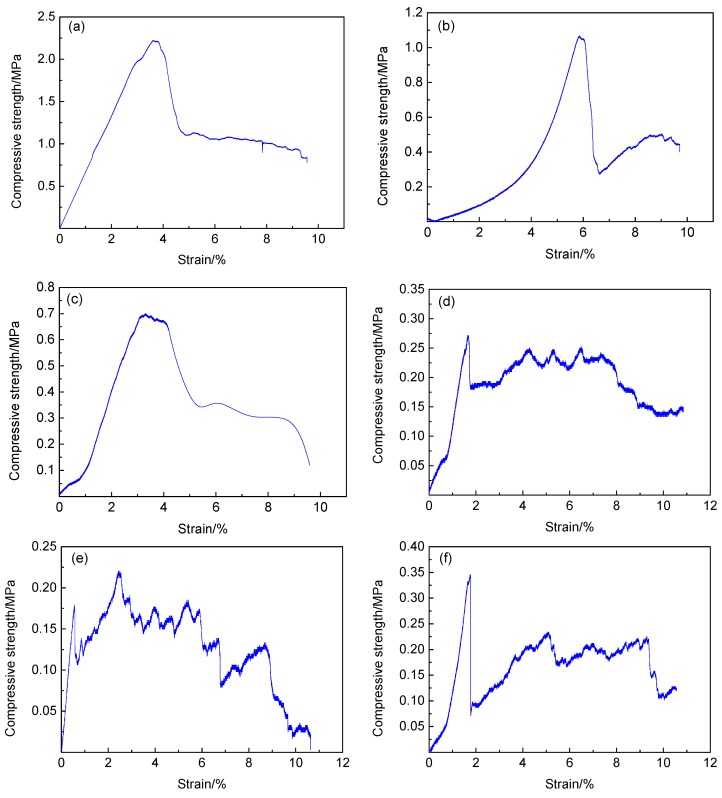
Stress-strain curves of samples under uniaxial compression, (**a**) stress-strain curve of sample B3, (**b**) stress-strain curve of sample B1, (**c**) stress-strain curve of sample D1, (**d**) stress-strain curve of sample A2, (**e**) stress-strain curve of sample A3 and (**f**) stress-strain curve of sample A1.

The uniaxial compressive strength of each sample is shown in Table 2, and the stress–strain curve of some samples is shown in Figure 4. According to the data in the table, the uniaxial compressive strength of the foamed geopolymer materials of different components prepared by this process varies greatly, with compression strength ranging from 0.221–2.221 MPa. The changes in components greatly influence the compressive strength of the materials.

Figure 4 shows that the uniaxial compression stress–strain curve of the sample has two major trends. The first type of curve is shown in Figure 4a–c. The peak strength of this type of stress–strain curve is evident, and only one peak appears in the entire compression process. The sample corresponding to this type of curve is elastic deformation at the initial stage of loading. The sample gradually enters the plastic deformation stage with increased load. In this stage, cracks begin to appear in the sample and gradually penetrate until the peak strength is reached. After the peak strength is exceeded, the deformation of the sample continues to increase, and a certain residual strength, which can continue to bear part of the load, is obtained.

The second type of curve is shown in Figure 4d,e. The peak strength is not evident, and multiple peaks exist. The load borne by the sample fluctuates within a certain range with increased deformation. In the initial stage of loading, the peak value of compressive strength is low, and the first peak value appears in place of the small deformation. This phenomenon is due to the many initial defects in the sample. Once loaded, the cracks rapidly penetrate along the initial defects, leading to sample failure. After the first peak value appeared under small strain, multiple peaks appeared with gradually increased deformation, and the average stress level in the sample remained basically unchanged within a relatively long deformation range. In this process, the pore structure in the sample is gradually destroyed. In addition, the sample continues to deform under constant stress level, and a long stress platform section appears. In this stress platform section, the sample can continuously undergo plastic deformation to absorb energy under the constant stress level. This property is crucial to the material’s resistance to dynamic impact, thereby enabling the material to absorb energy through its own deformation under external load; thus, the internal structure is protected from impact stress.

Figure 4f shows the combination of the first and second type curves, with a clear stress peak at approximately 1.6% of the strain, i.e., 0.34 MPa. After the peak value, the stress decreased rapidly and then the stress gradually increased at the point of approximately 2% strain until the strain was approximately 4%. Then, the stress recovered to 0.2 MPa. With further increased deformation, the stress platform stage appeared, until the strain was approximately 9.3%. The average stress remained unchanged at 0.2 MPa, and when the strain reached 10%, the sample was completely destroyed. The stress level is maintained at approximately 0.2 MPa within the strain range of 2–10%, which is the continuous energy absorption section of the sample.

### 3.2. Mineral Composition Characteristics of Foamed Geopolymer

The reaction process of geopolymers is a process of repolymerization after depolymerization. During this process, the fly ash spherical particles are dissolved, the Al-O and Si-O bonds are destroyed, and new non-crystalline gelatinous substances are formed [46,47,48,49,50,51,52,53,54].

**Figure 5:**
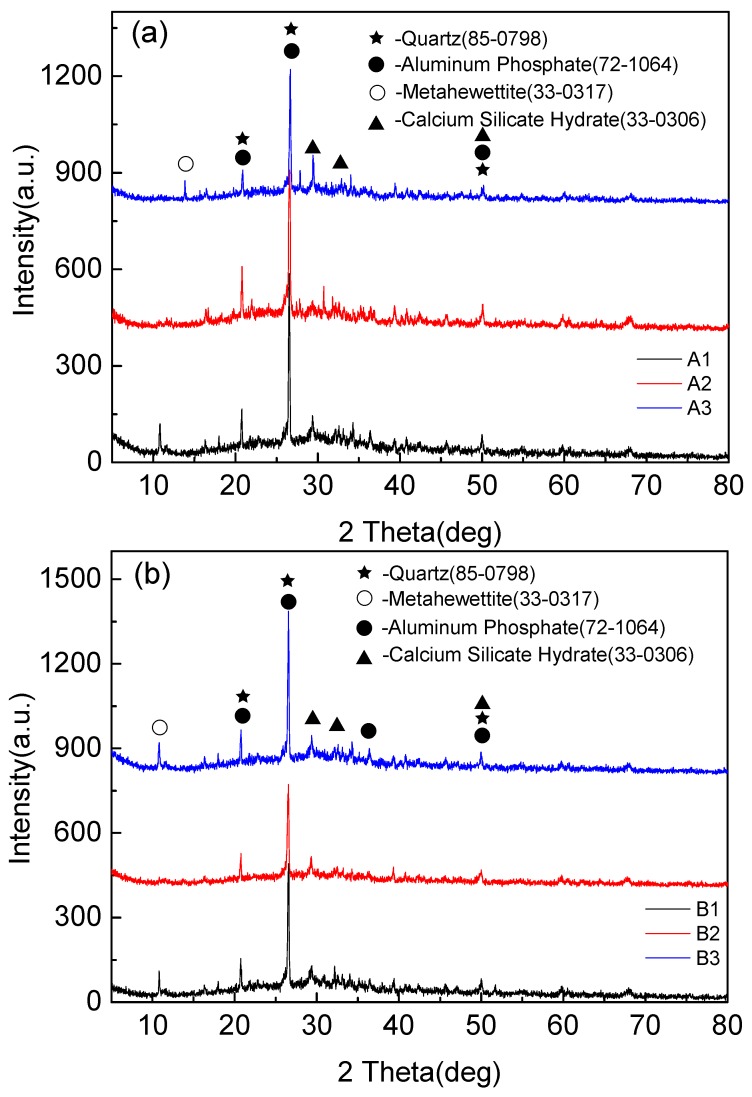

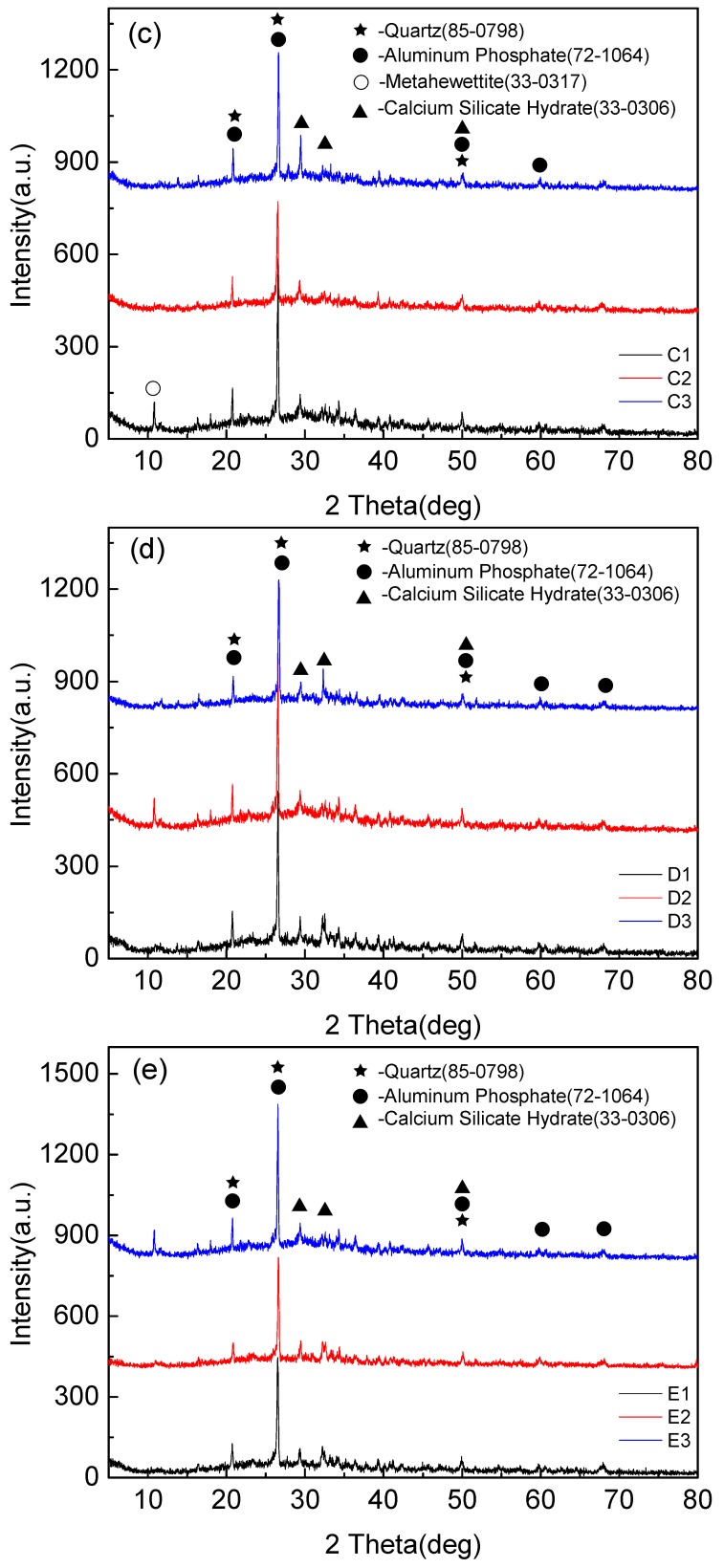
XRD diffraction pattern of foamed geopolymer based on fly ash. (**a**) XRD patterns of samples A1, A2, A3, (**b**) XRD patterns of samples B1, B2, B3, (**c**) XRD patterns of samples C1, C2, C3, (**d**) XRD patterns of samples D1, D2, D3, and (**e**) XRD patterns of samples E1, E2, E3.

Figure 5 shows that the mineralogical compositions of fly-ash-based foamed geopolymers of different components are slightly different, primarily Quartz, Aluminum Phosphate, Calcium Silicate, and Metahewettite. Quartz is the residue of fly ash and has low reactivity. Aluminum Phosphate is the crystal in the hydration products of cement hydration, and Calcium Silicate hydrates are the hydration products of Portland cement clinker during cement hydration reaction. Metahewettite is the product of the impurity in fly ash (MgO, Fe_2_O_3_) and Si-O bond in fly ash under the action of alkali activator.

The XRD patterns showed that the main components of the polymerization products were amorphous structures. It needs to be explained that obvious diffraction peaks appeared near 27 degrees in the spectrum, but this product was not the result of the polymerization reaction, but the quartz phase existing in the raw material fly ash, which is introduced by fly ash. There was a small amount of phosphate in the tap water for the test, and the aluminum phosphate produced by the ordinary Portland cement particles after absorbing the phosphate in the water. Metahewettite was formed by Cao in fly ash and cement under strong alkali environment.

### 3.3. Pore structure Characteristics

To further understand the pore structure characteristics and chemical reactions of the materials, the SEM images of the samples were compared and analyzed.

**Figure 6:**
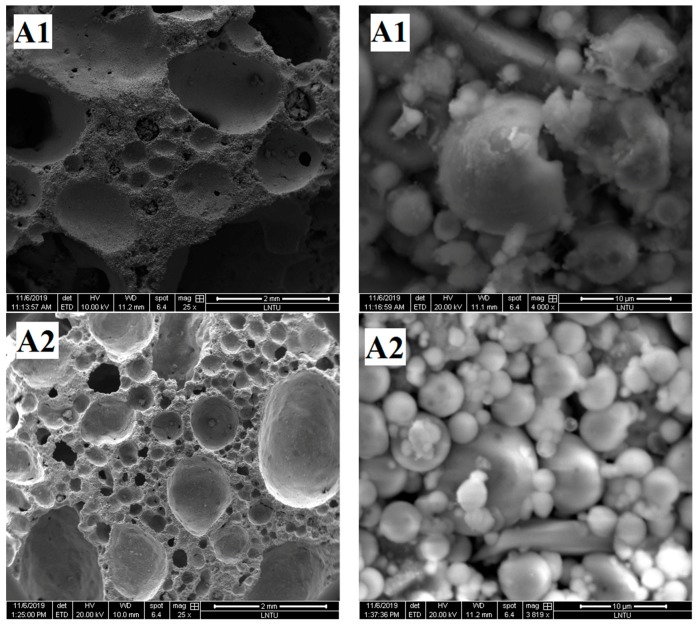

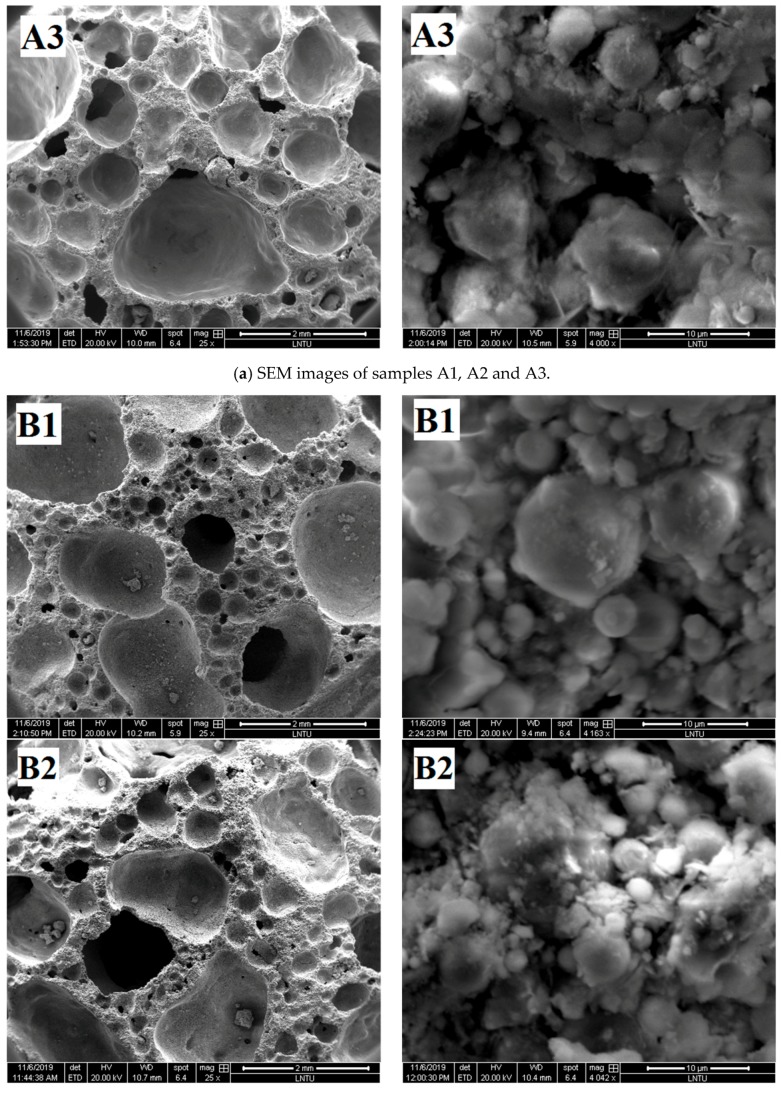

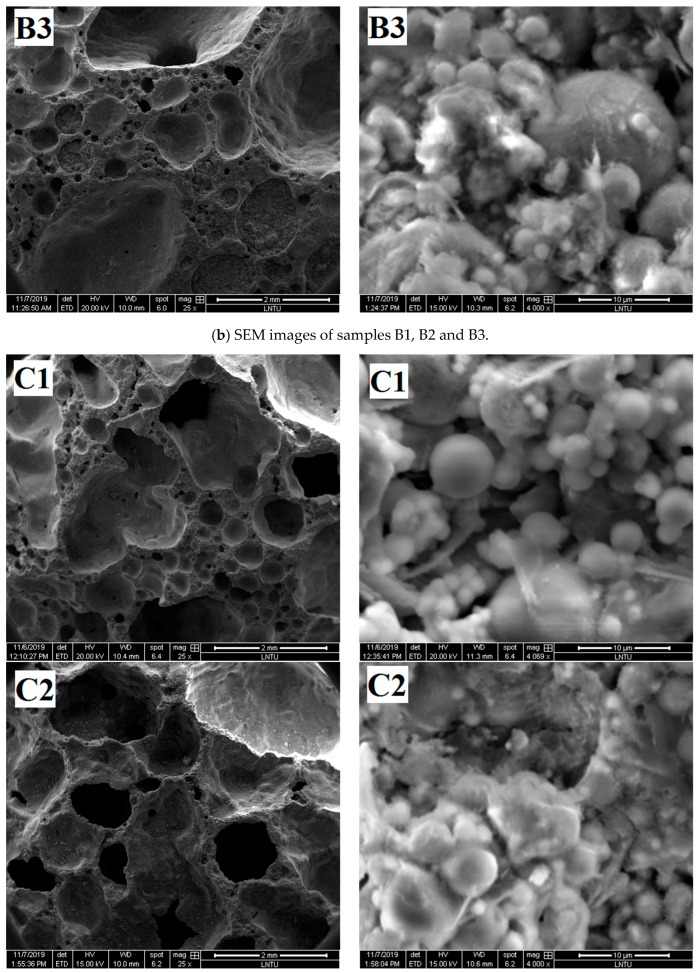

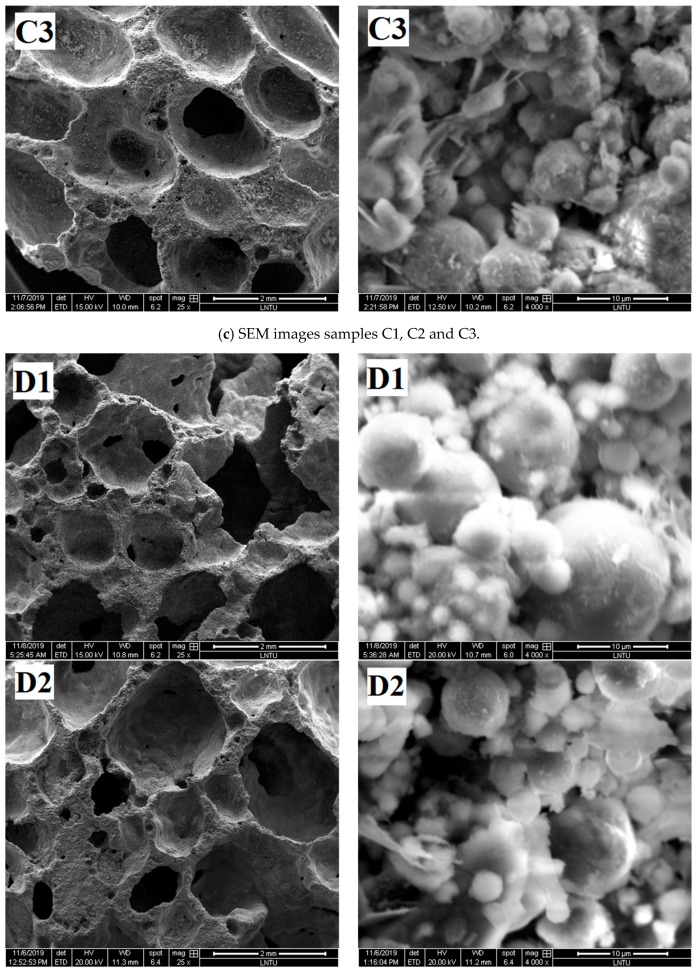

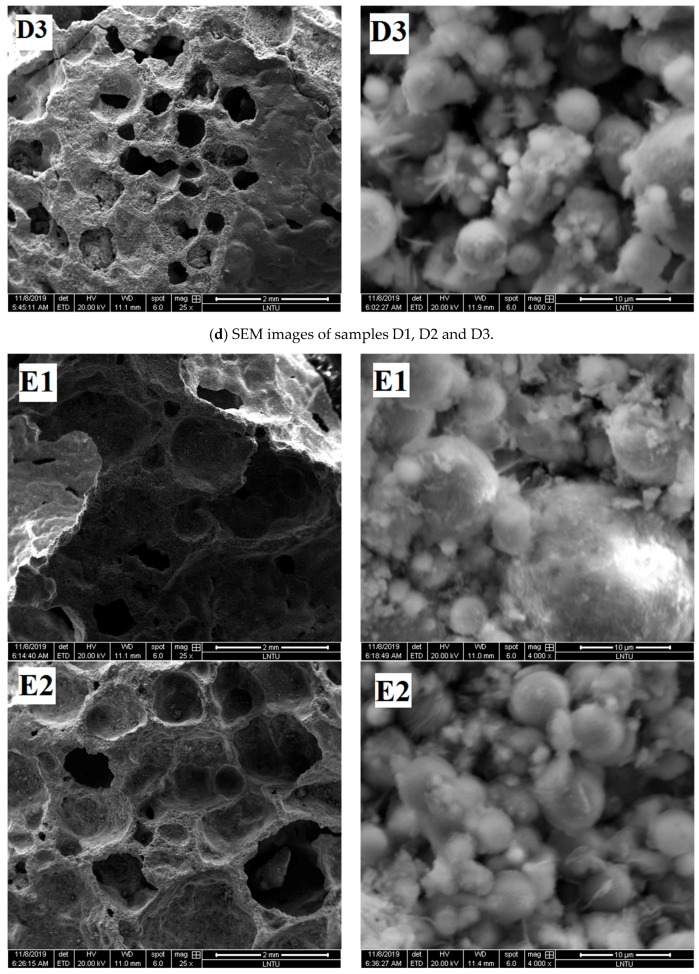

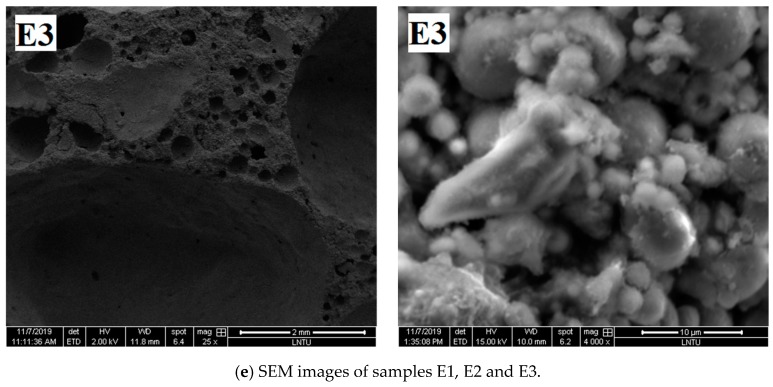
SEM images of samples.

The pore structure graphs and micrographs of group A samples are shown in Figure 6a. Figure 6a shows that the A2 sample has agglomeration of fly ash spherical particles.

The agglomeration of fly ash easily occurs after moisture absorption, which prevents the external alkali solution from entering and reduces the polymerization degree of fly ash. Few gelled polymers exist between spherical particles in Samples A1, A2, and A3, which cannot form an effective and firm connection, resulting in low density and compressive strength of foamed polymers.

Figure 6b shows that when the content of H_2_O_2_ is different, the degree of polymerization of the obtained samples is also different. Relatively few gelatinous substances exist between fly ash particles of B1 samples, but the fly ash particles of B2 and B3 samples are almost filled with amorphous gelatinous substances. Correspondingly, according to the analysis in Section 3.1.2, the compressive strength of B2 and B3 samples is also greater than that of B1 samples. According to the analysis, with increased H_2_O_2_ content, the mixed slurry continuously has bubbles and move in the grout prior to the initial setting, thereby promoting the full flow contact of each component in the slurry and increasing the reaction degree of fly ash ball particles. On the contrary, when the H_2_O_2_ content is relatively small, the mixed slurry produces few bubbles prior to the initial setting, resulting in almost static slurry. The gelatinous substance produced by the first reaction is wrapped around the fly ash, preventing the ion exchange between the inside and outside of the coating. Moreover, the polymerization reaction cannot further enter the interior, resulting in a small quantity of polymerization material, which reduces the strength of the sample.

Figure 6c shows that with gradually increased foam-stabilizer content, the pore diameter in the polymer transforms from extremely uneven to uniform. The pore diameter becomes uniform, and the solid parts, such as pore wall and pore ridge, become thin. With increased content of the foam-stabilizing agent, the degree of destruction of fly ash spherical particles gradually increases. Only parts of the fly ash particles in the C1 sample are surrounded by gelation, most fly ash particles in the C2 sample are surrounded by gelation, and almost all fly ash particles in the C3 sample are surrounded by gelation. The addition of foam-stabilizing agent contributes to the uniform distribution of pore diameter, but at the same time, the pore wall becomes thin, thereby leading to decreased sample strength.

Figure 6d shows that with increased content of sodium silicate, the reaction degree and compressive strength of the obtained sample increase. D1 sample has many defects in the pore structure and has serious hole wall defects. From the microscopic point of view, the surface integrity of fly ash spherical particles is relatively high, indicating that the reaction degree of Al-O and Si-O bonds in fly ash was relatively low and few gelling substances surrounding the particles. The pore structure integrity of D2 sample was improved, the small particles were surrounded by gelatinous substances, and the gel material between the large particles was insufficient. The pore diameter of the D3 sample is smaller than that of the D1 and D2 samples, and the large and small particles of the sample are surrounded by the gelling substances. The degree of participation of fly ash in the polymerization reaction is high; thus, the strength of the geopolymer formed is also high.

Figure 6e shows that with increased NaOH content, the pore structure integrity of the sample is gradually improved, and the degree of fly ash participating in the polymerization reaction increases. The pore wall defects of E1 sample are serious, holes exist on the pore wall, and pore edges are limited. The microscopic figure shows that the surface of the large fly ash particles is relatively smooth and not seriously damaged, and a small amount of gelatinous substance is generated on the surface. The fly ash spherical particles of the E2 sample are almost bonded by the gelatinous substance, and the surface of the globular particles is gray and lusterless, indicating that it is corroded by alkaline activator and that the reaction degree is improved. The hole structure of the E3 sample is a regular circular closed hole structure, which is conducive to bear the load. More gelatinous substances exist between the fly ash particles, and the sample formed has good integrity.

### 3.4. Analyses of Emissions Reduction and Cost, Energy Savings

Under the overall strategic layout of sustainable development implemented by the Chinese government, the greenhouse effect and global warming have prompted the people to actively develop low-carbon cement to replace ordinary Portland cement. In the 1990s, the world produced 1 billion tons of cement, resulting in 1 billion tons of CO_2_, which account for 7% of the world’s total CO_2_ emissions. Every 1 t of cement produced results in 1 t CO_2_ [55]. For fly-ash-based foamed geopolymer, the CO_2_ emission from the production of 1 t of fly-ash-based foamed geopolymer is approximately 0.09 t given that no high-temperature treatment is required in the production process. Compared with ordinary Portland cement, producing the same quality of foamed geopolymer produces only 9% of the CO_2_ emissions of cement, thereby greatly reducing greenhouse gas emissions [56,57,58].

Most industrial wastes contain aluminosilicate, such as kaolin, rock, fly ash, and other industrial wastes, which have been successfully used in the preparation of geopolymers. Therefore, the preparation materials of foamed geopolymers are widely available and low-cost. According to statistics, the ex-factory price of Portland cement is $1200–3200/t, and the production price of fly-ash-based foamed geopolymers is $200–700/t [59]. Here is one thing that needs to be made clear, the cost of activators in the experiment has not been taken into account. This is also one of the shortcomings in the current geopolymer manufacturing process, so further exploration of cheaper activators is needed in the future.

In addition, as the production process of cement requires high temperature treatment process of “two grinding and one burning,” the energy consumption of cement production is extremely high, at approximately 3200–3500 MJ/t, but the energy consumption of fly-ash-based foamed geopolymer is only 800–1000 MJ/t.

## 4. Conclusions

A type of fly-ash-based foamed geopolymer, which is suitable for goaf filling is prepared. This material showed good bearing capacity, environmental friendliness, energy saving, and low CO_2_ emission. Foamed geopolymer based on fly ash, H_2_O_2_, sodium stearate, sodium metasilicate, and NaOH was prepared. Uniaxial compression test results showed that the fly-ash-based foamed geopolymer prepared by this process presented brittle failure under uniaxial compression condition. Cracks appeared in the sample at the early loading stage, and cracks gradually broke through with increased load. The sample was also divided into several vertical bar shapes. The failure of foamed geopolymer was primarily related to the “local large pore” in the sample and the bearing “weak zone” or “weak surface” formed by various initial defects. The compressive strength of the samples with different component contents was significantly different. The test results showed that the compressive strength of the sample was between 0.221 and 2.221 MPa.

The analysis of XRD experiment indicates that quartz was the residue of fly ash in the reaction product, Aluminum Phosphate is the crystal in the hydration products of cement hydration, and Calcium Silicate hydrates were the hydration products of Portland cement clinker during the cement hydration reaction.

When the contents of each component differed, the reaction degree of the obtained geopolymer also differed. Relatively few gelatinous substances exist between the spherical particles of the fly ash of some samples, but the gaps around the fly ash particles of the other samples were almost filled with amorphous gelatinous substances, thereby resulting in high density of foamed geopolymer and corresponding high compressive strength.

Finally, the CO_2_ emission, energy consumption, and economy in the production of fly-ash-based foaming geopolymer were analyzed in detail. Compared with traditional foamed concrete, foamed geopolymer had the advantages of low carbon emission, low energy consumption, and low cost. In the future, it will have a wide application potential in goaf filling.

## Figures and Tables

**Table 1:** Mixture proportion of fly-ash-based foamed geopolymer composites.

Mix No.	Fly Ash/g	Cement/g	Sodium Metasilicate/g	Water/mL	H_2_O_2_/mL	NaOH/g	Sodium Stearate/g
A1	150	350	11.7	215	35	35	0
A2	75	425	11.7	215	35	35	0
A3	0	500	11.7	215	35	35	0
B1	150	350	11.7	215	20	35	0
B2	150	350	11.7	215	35	35	0
B3	150	350	11.7	215	50	35	0
C1	150	350	11.7	215	35	35	0
C2	150	350	11.7	215	35	35	0.12
C3	150	350	11.7	215	35	35	0.25
D1	150	350	8.88	215	35	35	0
D2	150	350	11.7	215	35	35	0
D3	150	350	14.6	215	35	35	0
E1	150	350	11.7	215	35	20	0
E2	150	350	11.7	215	35	27	0
E3	150	350	11.7	215	35	35	0

**Table 2:** Physical and mechanical parameters of the samples.

NO.	Mass/g	Volume/cm^3^	Density/g/cm^3^	Compressive Strength/MPa
A1	406	1000	0.406	0.348
A2	393	1000	0.393	0.274
A3	388	1000	0.388	0.221
B1	480	1000	0.48	1.066
B2	446	1000	0.446	2.221
B3	428	1000	0.428	2.219
C1	417.5	1000	0.4175	0.598
C2	411.5	1000	0.4115	0.452
C3	424	1000	0.424	0.391
D1	440	1000	0.44	0.700
D2	417.5	1000	0.4175	0.977
D3	410	1000	0.41	1.246
E1	409	1000	0.409	1.466
E2	438.5	1000	0.4385	1.864
E3	492	1000	0.492	2.019
